# Embryologic and Developmental Origins of Gastroschisis: A Scoping Review of Historical and Contemporary Theories

**DOI:** 10.3390/children13020270

**Published:** 2026-02-14

**Authors:** Mohamad Abi Nassif, Emrah Aydin, Jose L. Peiro

**Affiliations:** 1 The Center for Fetal and Placental Research, Cincinnati Fetal Center, Division of Pediatric General and Thoracic Surgery, Cincinnati Children’s Hospital Medical Center (CCHMC), Cincinnati, OH 45229, USA; mohamad.jamal.abi.nassif@cchmc.org (M.A.N.); jose.peiro@nyulangone.org (J.L.P.); 2 Division of Pediatric Gastroenterology, Hepatology and Nutrition, Cincinnati Children’s Hospital Medical Center (CCHMC), Cincinnati, OH 45229, USA; 3 Division of Maternal Fetal Medicine, Jackson Fetal Care, Department Obstetrics, Gynecology and Reproductive Sciences, Miller School of Medicine, University of Miami, Miami, FL 33136, USA; 4 NYULH Advanced Fetal Care Center, NYU Langone Health, New York, NY 10016, USA

**Keywords:** gastroschisis, embryology, abdominal wall defects, umbilical ring, prenatal diagnosis, ultrasound, magnetic resonance imaging, placental pathology, developmental mechanisms

## Abstract

**Highlights:**

**What are the main findings?**

- Fourteen embryologic theories of gastroschisis can be systematically organized into 4 mechanistic categories: mesodermal and ventral body wall folding abnormalities, vascular disruption, umbilical ring and extraembryonic attachment defects, and integrated multifactorial developmental models.
- Historical theories describe complementary components of overlapping developmental pathways rather than mutually exclusive mechanisms, reflecting the absence of a single unifying embryologic explanation.
- Embryologic, placental, and experimental evidence converges in a multifactorial process involving mesodermal vulnerability, localized vascular instability, and structural susceptibility at the umbilical abdominal wall junction, providing a biologic explanation for the consistent right-sided predominance and phenotypic variability of gastroschisis.

**What are the implications of the main findings?**

- Gastroschisis is best understood as a spectrum of periumbilical developmental disturbances rather than a single isolated abdominal wall defect.
- An integrated embryologic framework supports more precise prenatal interpretation, risk stratification, counseling, and classification of simple, complex, and closing forms of gastroschisis.
- Future research should focus on molecular, vascular, and biomechanical interactions at the umbilical ring to refine mechanistic understanding and inform preventive and therapeutic strategies.

**Abstract:**

Background/Objectives: Gastroschisis remains one of the most debated congenital abdominal wall defects with respect to its embryologic and developmental origins. Despite decades of investigation, no consensus exists regarding a single causative mechanism, and competing hypotheses variably explain laterality, bowel injury, and closing variants. This scoping review aims to synthesize historical and contemporary embryologic theories of gastroschisis and integrate them into a coherent developmental framework with direct relevance to prenatal assessment and clinical interpretation. Methods: A structured literature search was conducted in PubMed, Web of Science, and Scopus from inception through December 2025. Studies proposing original embryologic mechanisms or providing primary experimental, placental, or developmental evidence were included. Eligible publications were qualitatively synthesized and classified according to evidence strength as historical descriptive, experimental, placental pathology, or integrative synthesis. Embryologic theories were organized into mechanistic categories based on affected structures, developmental timing, and proposed pathophysiology. Results: Twenty-six publications met inclusion criteria, yielding fourteen distinct embryologic theories. These were categorized into four mechanistic categories: mesodermal and ventral body wall folding abnormalities, vascular disruption models, umbilical ring and extraembryonic attachment defects, and integrated multifactorial developmental concepts. No single mechanistic category alone consistently accounted for right-sided predominance, variability in bowel injury, and the occurrence of closing variants. Conclusions: Gastroschisis is best understood as a spectrum of periumbilical developmental disturbances arising from interacting mesodermal, vascular, and biomechanical factors. An integrated embryologic framework improves interpretation of dynamic prenatal imaging findings, supports refined risk stratification and counseling, and provides a biologic foundation for future translational research.

## 1. Introduction

Gastroschisis is a congenital abdominal wall defect characterized by extrusion of the fetal intestine through a paraumbilical opening, most commonly located to the right of the umbilical cord insertion. In contrast to omphalocele, the defect lacks a covering membrane and is frequently associated with variable degrees of bowel inflammation, dysmotility, and intestinal atresia, resulting in a broad clinical spectrum ranging from simple defects to complex and closing forms with substantial morbidity [1,2]. Despite advances in prenatal imaging, neonatal care, and surgical management, the embryologic and developmental origins of gastroschisis remain incompletely resolved [3].

Over the past several decades, multiple hypotheses have been proposed to explain the pathogenesis of gastroschisis. Early theories emphasized intrinsic abnormalities of mesodermal development and ventral body wall folding [4,5,6,7,8,9], whereas subsequent models focused on vascular disruption, ischemic injury, or involution of embryonic vessels [10,11,12,13,14,15,16]. More recent concepts have shifted attention toward defects of the umbilical ring and extraembryonic attachments, reframing gastroschisis as a localized periumbilical developmental disturbance rather than a generalized failure of abdominal wall closure [17,18,19,20]. These hypotheses have often been presented as competing explanations, contributing to fragmented literature and persistent lack of consensus [3].

Previously published mechanistic explanations for gastroschisis can be broadly grouped into three categories. The mesodermal and ventral body wall folding abnormality model proposes defective lateral folding or mesodermal thinning during early embryogenesis, resulting in a localized failure of ventral body wall closure adjacent to the umbilicus [4,5,6,7,8,9]. Vascular disruption models attribute gastroschisis to localized ischemic injury caused by abnormal development, regression, or occlusion of embryonic abdominal wall vessels, leading to secondary tissue loss [10,11,12,13,14,15,16]. Umbilical ring and extraembryonic attachment defect theories emphasize structural weakness or abnormal remodeling at the umbilical ring, allowing herniation of midgut structures through a periumbilical defect [17,18,19,20].

Recent reviews underscore this uncertainty. Muniz et al. highlighted the absence of agreement on a single pathogenic mechanism and emphasized that existing theories variably account for laterality, bowel injury, and clinical heterogeneity [21]. Such observations suggest that continued pursuit of a unifying singular cause may be inherently limiting. Instead, a framework capable of integrating multiple developmental processes may be more consistent with the accumulated evidence [2,12,20].

Epidemiologic and placental studies further support a multifaceted developmental process. Gastroschisis exhibits a consistent right-sided predominance and has been associated with placental vascular abnormalities, including thrombotic vasculopathy and delayed villous maturation, implicating vascular and placental contributions without identifying a single reproducible vascular insult [10,11,16,22,23]. Similarly, reports of placental mesenchymal dysplasia in association with gastroschisis further support the involvement of abnormal fetoplacental vascular and stromal development in the pathogenesis of the defect [24]. At the same time, prenatal imaging studies demonstrate dynamic changes in bowel appearance and defect morphology over gestation, particularly in complex and closing variants, which are not adequately explained by isolated mesodermal or vascular models alone [2,25].

From a clinical standpoint, the absence of a coherent embryologic framework limits interpretation of prenatal ultrasound and magnetic resonance imaging findings and complicates counseling regarding disease severity, progression, and surgical complexity [2,25]. Recent work identifying prenatal markers associated with complex gastroschisis highlights the need to contextualize imaging findings within a biologically plausible developmental model rather than viewing them as static anatomic abnormalities [25].

The objective of this review is to provide a structured, evidence-informed synthesis of historical and contemporary embryologic theories of gastroschisis. Using transparent selection criteria and evidence-informed classification, we aim to organize existing hypotheses into coherent mechanistic categories based on affected structures, developmental timing, and supporting evidence [2,3,4,12,20]. By integrating embryologic, placental, experimental, and prenatal imaging data, this review seeks to reconcile longstanding controversies and offer a unified developmental framework with direct relevance to prenatal assessment, risk stratification, and future translational research [2,3,12,20,25].

## 2. Materials and Methods

**Figure 1:**
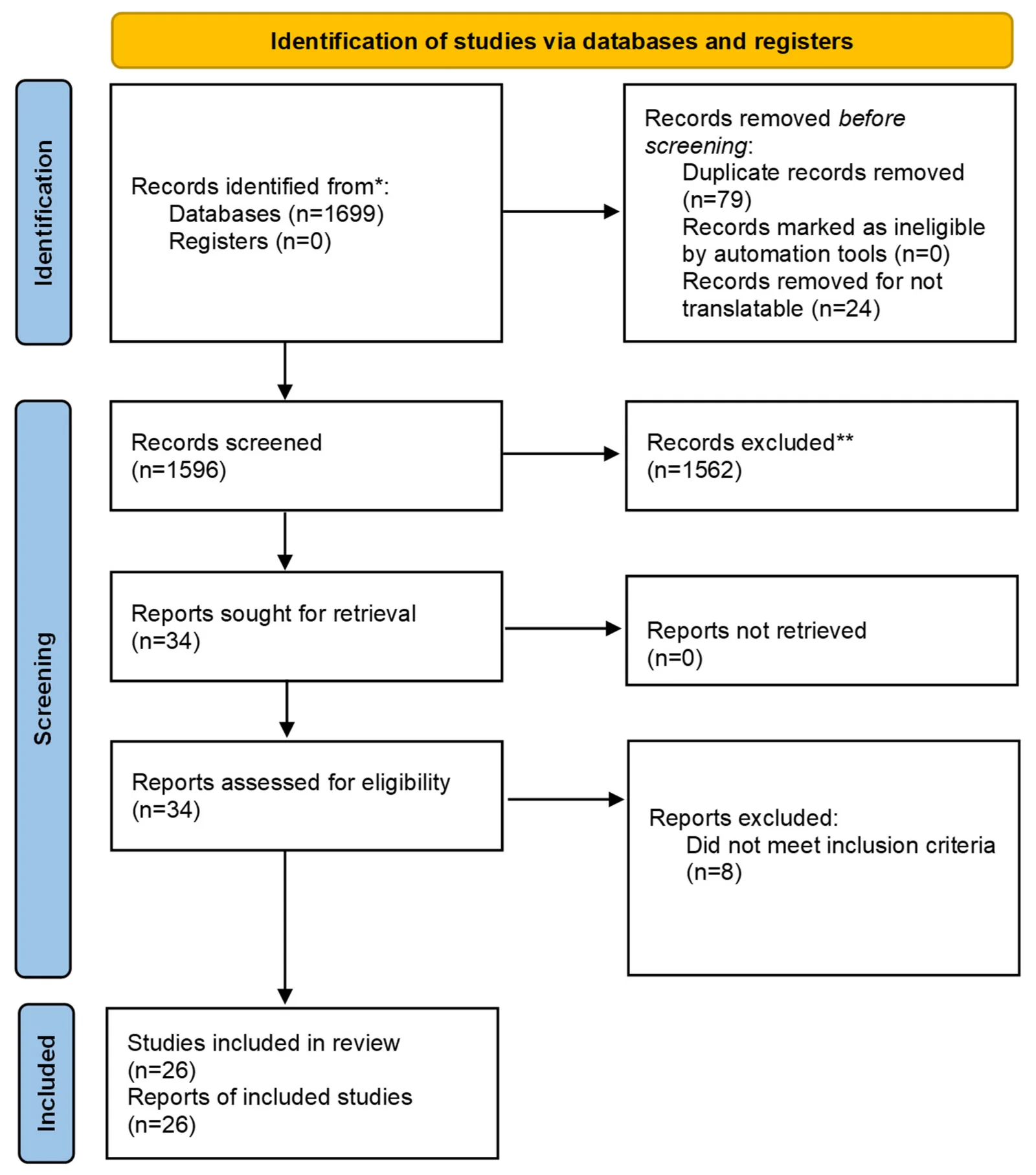
PRISMA 2020 flow diagram illustrating the identification, screening, eligibility assessment, and inclusion of studies for the structured systematic synthesis of embryologic theories of gastroschisis. * Records identified through database searching represent the total number of citations retrieved from electronic databases prior to duplicate removal and preliminary eligibility checks. ** Records excluded at the screening stage refer to studies removed after title and abstract evaluation because they did not address primary embryologic mechanisms, lacked mechanistic relevance, or focused solely on clinical management, epidemiology, or surgical techniques without developmental analysis.

This review was designed as a scoping review of embryologic and developmental theories of gastroschisis. Given the hypothesis-driven, heterogeneous, and predominantly descriptive nature of the available literature, the objective was a qualitative integration of mechanistic evidence rather than quantitative effect estimation. The methodology emphasizes transparency and reproducibility, following the principles of the PRISMA Extension for Scoping Reviews (PRISMA-ScR). Although the literature search followed a structured approach, the synthesis was narrative in nature, reflecting the historical characteristics of research in this field. The methodology therefore emphasizes transparency, reproducibility, and evidence informed synthesis, consistent with PRISMA 2020 guidance for Scoping Reviews of non-interventional literature and was prospectively registered with PROSPERO (CRD420261280180) (Figure 1). 

### 2.1. Literature Search Strategy

A comprehensive literature search was conducted in PubMed MEDLINE, Web of Science, and Scopus from database inception through December 2025. Search terms included gastroschisis, embryology, abdominal wall development, ventral body wall folding, umbilical ring, vascular disruption, placental pathology, and developmental mechanisms, combined using Boolean operators. No registers were searched. Reference lists of eligible studies and relevant reviews were manually screened to identify additional publications.

### 2.2. Study Identification and Screening

The database search yielded 1699 records. After removal of duplicate records (*n* = 79) and records excluded due to language barriers preventing reliable translation (*n* = 24), 1596 records underwent title and abstract screening. Of these, 1562 records were excluded because they focused exclusively on surgical technique, clinical outcomes, epidemiologic associations without mechanistic interpretation, or prenatal management without embryologic relevance.

Thirty-four full text reports were sought and successfully retrieved. Following full text assessment, 8 reports were excluded for failure to meet inclusion criteria, most commonly due to absence of original mechanistic hypotheses or reliance on secondary citation without primary evidence. Ultimately, 26 studies met predefined inclusion criteria and were included in the qualitative synthesis. The study selection process is summarized in the PRISMA 2020 flow diagram.

### 2.3. Eligibility Criteria

Studies were eligible for inclusion if they proposed an original embryologic or developmental mechanism for gastroschisis or provided primary experimental, placental, or developmental evidence directly relevant to its pathogenesis. Historically influential descriptions that shaped subsequent mechanistic theories were included even in the absence of modern experimental validation. Studies limited to surgical technique, isolated case reports, or purely descriptive clinical series without mechanistic interpretation were excluded.

### 2.4. Data Extraction

For each included study, data were extracted on year of publication, proposed embryologic mechanism, primary anatomic or developmental structure implicated, type of supporting evidence, and key limitations. Data extraction focused on mechanistic content rather than clinical outcomes, reflecting the objectives of the review.

### 2.5. Evidence Strength Classification and Descriptive Characterization

To support the systematic nature of the synthesis without applying inappropriate quantitative risk of bias tools, included studies were classified according to predefined evidence strength categories: historical descriptive, experimental, placental pathology, or integrative synthesis. In addition, the nature of the supporting evidence was assessed to contextualize the findings within each category. Historical descriptive studies were considered hypothesis generating, experimental and placental pathology studies were considered moderate strength mechanistic evidence, and integrative syntheses were evaluated based on coherence, consistency, and integration of multiple evidence domains. This adapted assessment provides methodological transparency while acknowledging the limitations inherent to embryologic hypothesis driven literature. Detailed classifications are provided in the Table 1. This classification was applied to transparently characterize the heterogeneity of evidence sources, consistent with the objectives of a scoping review, and does not constitute a formal risk of bias assessment.

### 2.6. Qualitative Synthesis and Classification Framework

Embryologic theories were synthesized qualitatively and organized into mechanistic categories using 3 predefined criteria: the primary developmental structure involved, the proposed timing of the developmental disturbance, and the conceptual localization of the defect within the umbilical abdominal wall continuum. Based on this framework, theories were grouped into mesodermal and ventral body wall folding abnormalities, vascular disruption models, umbilical ring and extraembryonic attachment defects, and integrated multifactorial developmental models. This classification was applied consistently across included studies and forms the basis for the comparative analysis presented in the Results.

## 3. Results

The included studies spanned more than 7 decades and represented heterogeneous evidence domains, including historical descriptive embryology, experimental and developmental biology studies, placental pathology analyses, and integrative theoretical syntheses. Based on predefined evidence strength criteria, studies were classified as historical descriptive, experimental, placental pathology, or integrative synthesis. No interventional or comparative studies were identified, consistent with the hypothesis driven nature of embryologic research in gastroschisis.

Qualitative synthesis identified 14 distinct embryologic theories. Using predefined classification criteria related to the primary affected structure, developmental timing, and localization of the defect, these theories were systematically organized into 4 mechanistic categories: mesodermal and ventral body wall folding abnormalities, vascular disruption models, umbilical ring and extraembryonic attachment defects, and integrated multifactorial developmental models. The distribution of theories and supporting evidence across categories is summarized in Table 1.

**Figure 2:**
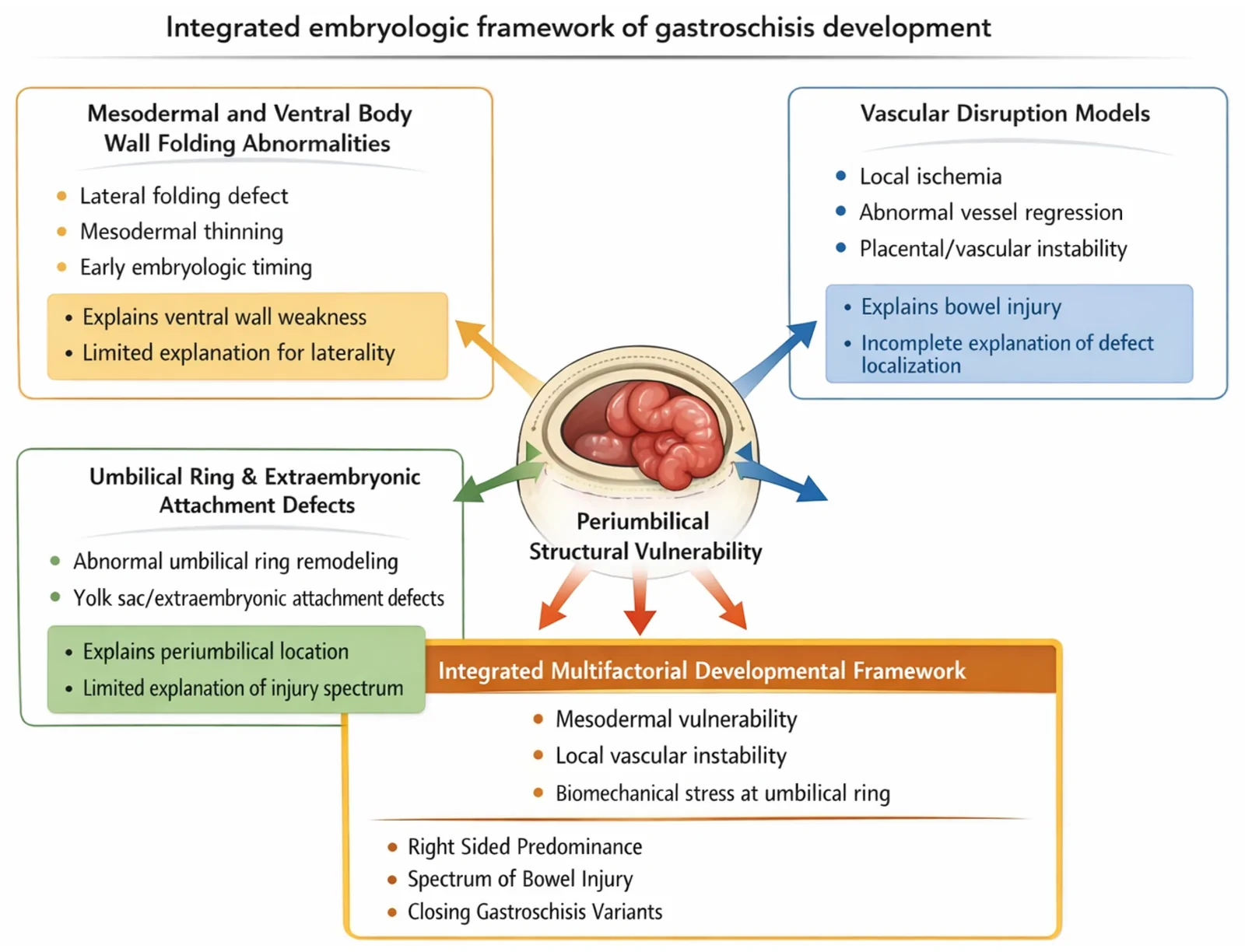
Integrated embryologic framework of gastroschisis development illustrating convergence of mesodermal, vascular, and umbilical ring-related mechanisms.

Mesodermal and ventral body wall folding theories primarily attributed gastroschisis to intrinsic abnormalities of early mesodermal development or failure of lateral body wall closure (Figure 2). These models explained defect formation but did not consistently account for right-sided predominance, progressive bowel injury, or the occurrence of closing variants. Vascular disruption models proposed localized ischemic injury secondary to embryonic or placental vascular compromise and provided plausible explanations for laterality and placental associations. However, no single vascular mechanism reproducibly explained the full phenotypic spectrum, particularly cases with progressive narrowing or late closure of the defect.

Theories centered on the umbilical ring and extraembryonic attachments localized the primary disturbance to the transitional zone between the umbilical cord and abdominal wall. These models accounted for the periumbilical location of the defect and offered mechanistic explanations for closing and vanishing gastroschisis. Nonetheless, when considered in isolation, they did not fully explain the variability in bowel injury severity or the frequent association with intestinal atresia.

Accordingly, integrated multifactorial models emerged as the only framework capable of explaining the full phenotypic spectrum of gastroschisis.

## 4. Discussion

This scoping review indicates that gastroschisis does not arise from a single embryologic defect but from interacting developmental disturbances affecting the periumbilical region. Historical and contemporary theories, when examined in isolation, each explain selected aspects of the condition but fail to account for its full anatomic and clinical spectrum [2,12,15,21,26]. When integrated, however, these models describe complementary components of a shared developmental continuum involving mesodermal vulnerability, vascular instability, and structural susceptibility at the umbilical abdominal wall junction.

Early mesodermal and ventral body wall folding theories correctly emphasized intrinsic tissue weakness during early embryogenesis and remain essential for understanding initial defect formation [3,4,5,6,7,8,9,13]. Their primary limitation lies in the inability to consistently explain right-sided predominance and progressive bowel injury. Vascular disruption models addressed these shortcomings by proposing asymmetric ischemic injury linked to embryonic or placental vascular compromise [10,14,16,22,23,24]. Although supported by placental pathology findings, purely vascular explanations have struggled to identify a single reproducible event capable of explaining defect localization, variability in bowel injury, and late narrowing or closure of the defect [22,23,24].

Umbilical ring and extraembryonic attachment theories represent a critical conceptual advance by localizing the defect to the transitional zone between the umbilical cord and abdominal wall [17,18]. These models explain the consistent periumbilical location of gastroschisis and provide a biologically plausible mechanism for closing and vanishing variants. However, when considered alone, they do not fully account for the severity and heterogeneity of bowel injury, particularly in complex cases with associated intestinal atresia. Integrated multifactorial models emerged as the only framework capable of explaining the full phenotypic spectrum of gastroschisis.

Based on synthesis of these published mechanisms, we propose an integrated multifactorial developmental hypothesis incorporating mesodermal vulnerability, localized vascular instability, and structural susceptibility at the umbilical ring. This integrative framework is not presented as a previously established model, but rather as an author-derived synthesis intended to reconcile observations reported across experimental, placental, and prenatal imaging studies, which collectively suggest a dynamic developmental process rather than a static anatomic defect.

Integrated multifactorial developmental models reconcile these limitations by incorporating mesodermal susceptibility, vascular instability, and biomechanical constraint at the umbilical ring. Importantly, only integrated models consistently explain 3 defining features of gastroschisis: right-sided predominance, variability in bowel injury, and the occurrence of closing variants. Purely mesodermal models cannot account for progressive defect narrowing, while purely vascular models inadequately explain cases without clear ischemic markers. In contrast, an integrated framework allows for dynamic interaction among developmental processes over time, aligning with observed prenatal progression and postnatal heterogeneity.

Recent literature further supports this interpretation. Muniz et al. emphasized the lack of consensus on gastroschisis pathogenesis and highlighted the limitations of single mechanism theories, indirectly supporting the need for an integrative developmental framework [21]. Similarly, Caldas et al. identified prenatal ultrasound markers associated with complex gastroschisis, including progressive bowel dilation and wall thickening, findings that are difficult to reconcile with static mesodermal defects or isolated vascular insults [25]. Within an integrated model, these markers may reflect evolving biomechanical constraint and secondary vascular compromise at the umbilical ring, providing a biologically plausible explanation for disease progression and closing variants.

### 4.1. Clinical and Translational Implications

Reframing gastroschisis as a spectrum of periumbilical developmental disturbances has direct clinical implications. Prenatal ultrasound and magnetic resonance imaging findings should be interpreted dynamically rather than as static anatomic abnormalities. Progressive bowel dilation, reduced bowel mobility, or changes in defect morphology over gestation may indicate increasing biomechanical constraint or vascular compromise, signaling higher risk for complex or closing gastroschisis. Integrating embryologic mechanisms into prenatal assessment may therefore improve risk stratification, counseling, and anticipatory surgical planning.

This framework also provides a biologic basis for classification of disease severity. Simple gastroschisis may reflect limited periumbilical vulnerability, whereas complex and closing forms likely arise from combined mesodermal, vascular, and biomechanical disturbances. Although prospective validation is required, such integration offers a rational approach to aligning embryologic mechanisms with clinical phenotypes.

### 4.2. Limitations and Future Directions

This synthesis is limited by the predominantly descriptive and hypothesis generating nature of the available literature and by inherent constraints in studying human embryologic development. Nevertheless, structured organization of heterogeneous evidence clarifies areas of convergence and highlights critical gaps. Future research should prioritize molecular characterization of mesodermal development, detailed placental vascular analysis, and biomechanical modeling of stress at the umbilical abdominal wall junction. Advances in prenatal imaging and computational modeling may further refine mechanistic understanding and support translation into improved clinical management.

## 5. Conclusions

This scoping review demonstrates that gastroschisis cannot be adequately explained by any single embryologic mechanism. Instead, the available historical, experimental, placental, and developmental evidence supports a multifactorial developmental process centered on vulnerability of the periumbilical region. Mesodermal susceptibility, localized vascular instability, and biomechanical constraint at the umbilical abdominal wall junction interact dynamically to produce the characteristic laterality, bowel injury spectrum, and closing variants observed clinically.

By organizing fourteen embryologic theories into coherent mechanistic categories, this review reconciles previously competing hypotheses within a unified developmental framework. This integrated perspective provides a biologically plausible explanation for the heterogeneity of gastroschisis and shifts interpretation away from static anatomic defects toward dynamic developmental processes.

Clinically, adoption of an integrated embryologic framework supports more nuanced interpretation of prenatal imaging, improved risk stratification, and more informed counseling regarding disease progression and surgical complexity. Future advances will depend on integrating molecular embryology, placental pathology, biomechanical modeling, and longitudinal prenatal imaging to refine mechanistic understanding and translate developmental insights into improved clinical care.

## Figures and Tables

**Table 1:** Summary of Embryologic Theories of Gastroschisis and Supporting Evidence.

No	Theory/Model	Primary Proponent (Year)	Proposed Mechanism	Mechanistic Category	Primary Evidence Type	Key Strengths	Principal Limitations
1	Failure of lateral body wall folding	Duhamel (1963) [4]	Incomplete ventral body wall closure due to mesodermal defect	Mesodermal and body wall folding	Historical descriptive	Explains early defect formation	Does not explain right-sided predominance or bowel injury
2	Abnormal mesodermal differentiation	Shaw (1975) [5]	Localized mesodermal thinning adjacent to umbilicus	Mesodermal and body wall folding	Historical descriptive	Highlights intrinsic tissue vulnerability	Lacks experimental or placental validation
3	Failure of yolk sac incorporation	Hoyme et al. (1981) [10]	Persistence of extraembryonic structures weakens abdominal wall	Umbilical ring and extraembryonic attachment	Historical descriptive	Accounts for periumbilical localization	Limited ability to explain progressive bowel damage
4	Rupture of herniated bowel	Moore (1953) [1]	Physiologic herniation with secondary rupture	Mesodermal and body wall folding	Historical descriptive	Historically influential	Largely refuted by modern embryology
5	Right umbilical vein involution	De Vries (1980) [7]	Asymmetric vascular regression causing ischemia	Vascular disruption	Experimental/developmental	Explains right-sided predominance	Fails to explain closing variants
6	Omphalomesenteric artery disruption	Lubinsky (2014) [16]	Vascular compromise leading to localized necrosis	Vascular disruption	Placental pathology	Supported by placental vascular findings	No single reproducible vascular event identified
7	Abnormal placental perfusion	Feldkamp et al. (2007) [12]	Global placental insufficiency with focal abdominal effect	Vascular disruption	Placental pathology	Links epidemiologic risk factors	Mechanism remains indirect
8	Umbilical ring weakness	Rittler (2013) [18]	Structural vulnerability at umbilical abdominal junction	Umbilical ring and extraembryonic attachment	Historical descriptive	Explains periumbilical location	Does not explain bowel injury severity alone
9	Persistent extraembryonic attachment	Curry et al. (2000) [26]	Mechanical tethering at umbilical ring	Umbilical ring and extraembryonic attachment	Experimental	Accounts for defect localization	Limited human validation
10	Mechanical constriction model	Tibboel et al. (2012) [27]	Progressive constriction at umbilical ring	Umbilical ring and extraembryonic attachment	Experimental	Explains closing gastroschisis	Requires combination with other mechanisms
11	Placental vascular–mechanical interaction	Torfs et al. (1994) [11]	Combined vascular and structural effects	Integrated multifactorial	Placental pathology	Integrates epidemiology and anatomy	Heterogeneous evidence
12	Mesodermal–vascular interaction	Lubinsky (2014) [16]	Dual insult involving tissue and blood supply	Integrated multifactorial	Integrative synthesis	Explains laterality and injury	Largely theoretical
13	Dynamic periumbilical vulnerability	Muniz et al. (2024) [21]	Evolving mesodermal and vascular instability	Integrated multifactorial	Integrative synthesis	Highlights lack of single mechanism	Requires longitudinal validation

## Data Availability

The original contributions presented in this study are included in the article. Further inquiries can be directed to the corresponding author.

## Abbreviations

The following abbreviations are used in this manuscript:

MRI	Magnetic resonance imaging
PRISMA	Preferred Reporting Items for Systematic Reviews and Meta Analyses
US	Ultrasound
